# Laparoscopic sleeve gastrectomy as a bridge to colorectal cancer surgery for obese patients: a case report

**DOI:** 10.1186/s40792-024-02012-7

**Published:** 2024-09-09

**Authors:** Yume Minagawa, Manabu Amiki, Keisuke Yuki, Kazuharu Watanabe, Ichitaro Mochizuki, Yasuhiro Ishiyama, Yoshiaki Hara, Kazuhiro Narita, Yasumitsu Hirano

**Affiliations:** 1 Department of Surgery, Kawasaki Saiwai Hospital, 31-27 Omiya-Cho, Saiwai-Ku, Kawasaki-Shi, Kanagawa 212-0014 Japan; 2 Saitama Medical University International Medical Center, Hidaka City, Saitama Japan

**Keywords:** Bridge bariatric surgery, Colorectal cancer, Sleeve gastrectomy

## Abstract

**Background:**

Severe obesity greatly influences the difficulty of colorectal cancer surgery and has been reported to prolong operative time, increase the rate of laparotomy, and elevate increase postoperative complications. We investigated the efficacy of laparoscopic sleeve gastrectomy (LSG) for preoperative weight loss to ensure safe colorectal cancer surgery.

**Case presentation:**

A 51 year-old female with a body mass index of 43.5 kg/m^2^ was referred to our hospital due to a positive fecal occult blood test. She was diagnosed as having a laterally spreading tumor of the cecum by colonoscopy. Endoscopic submucosal dissection was attempted but proved difficult due to the size of the lesion and its proximity to the appendiceal orifice. We planned bariatric surgery prior to colorectal surgery, and she underwent LSG without any complications. Seven months after the LSG, she had lost 30.7 kg, and her final preoperative body mass index was 27.8 kg/m^2^. Single-incision laparoscopic ileocecal resection was then performed safely. The pathological diagnosis was adenocarcinoma in adenoma of the cecum, TisN0M0.

**Conclusion:**

LSG was effective in reducing visceral fat and making it possible to perform safe surgery for colorectal cancer in a severely obese patient.

## Background

The number of obese patients is increasing in Japan [1], and opportunities to treat these patients with colorectal cancer are also rising. In recent years, most colorectal cancer surgeries have been performed laparoscopically. A randomized controlled trial (JCOG 0404 study) conducted in Japan reported that the long-term prognosis of laparoscopic colorectal cancer surgery for obese patients with a body mass index (BMI) of 25 kg/m^2^ or more was worse than that of open surgery [2]. In obese patients, the intra-abdominal working space is narrower, making it difficult to obtain a good field of view and to protect tissues due to their fragility. Additionally, visceral fat affects the visualization of anatomic structures.

Weight loss is necessary for obese patients to safely undergo oncologic surgery, but significant weight loss is difficult to achieve with therapies such as diet and exercise. Metabolic and bariatric surgery is the most effective treatment for severe obesity and its associated medical problems [3]. Laparoscopic sleeve gastrectomy (LSG) has been the most commonly performed metabolic and bariatric surgery since 2014, with acceptable operative times, intraoperative blood loss, and perioperative complication rates [4]. Here, we report a case of LSG performed as a bridge procedure for early-stage colorectal cancer surgery.

## Case presentation

**Fig. 1:**
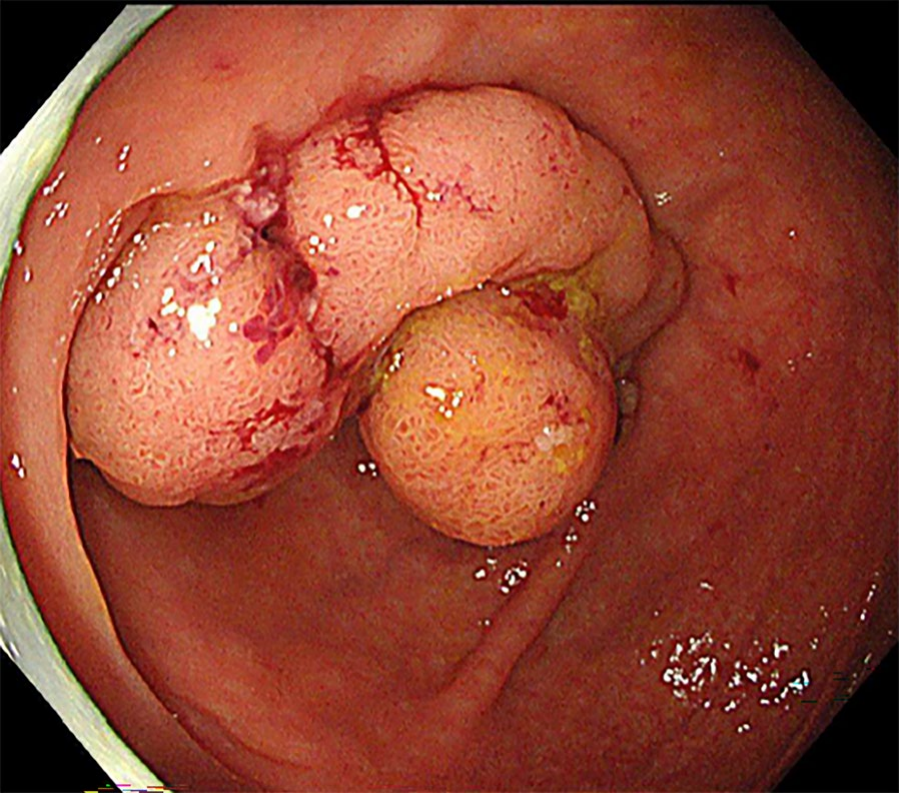
Endoscopic findings. Endoscopic image shows a laterally spreading tumor of the cecum

The patient was a 51 year-old female with hypertension managed with medication. She visited the gastroenterology department because of a positive fecal occult blood test and was diagnosed via endoscopy as having a laterally spreading tumor (25 mm × 25 mm) of the cecum. The polyp was pedunculated with smooth borders and no ulceration or hemorrhage. Blood investigations revealed a normal hemoglobin (13.4 g/dL). Tumor marker values were CEA 1.4 U/mL and CA19-9 2.0 ng/mL. The endoscopist judged this lesion to be amenable to endoscopic resection as an adenoma with a well-defined glandular ductal structure through assessment with magnifying endoscopy. Endoscopic submucosal dissection was attempted but was difficult due to the size of the lesion and its proximity to the appendiceal orifice. She was then referred to our department for surgical resection planning. Endoscopic findings showed a laterally spreading tumor of the cecum (Fig. 1), and biopsy results indicated a tubular adenoma. Computed tomography scans showed no metastasis. The preoperative diagnosis was adenoma or early adenocarcinoma of the cecum.

Her height was 154.9 cm, body weight was 104.5 kg, and BMI was 43.5 kg/m^2^. Given the technical difficulties anticipated due to severe obesity, we planned LSG seven months after the first consultation prior to resection of the primary tumor.

Although we recommended that she undergo early LSG, we had to wait seven months for the LSG due to her family circumstances. We judged that this period of time might be allowable because adenoma or early-stage cancer was assumed based on various findings. During the waiting period, she was on a restricted diet with nutritional guidance.

**Fig. 2:**
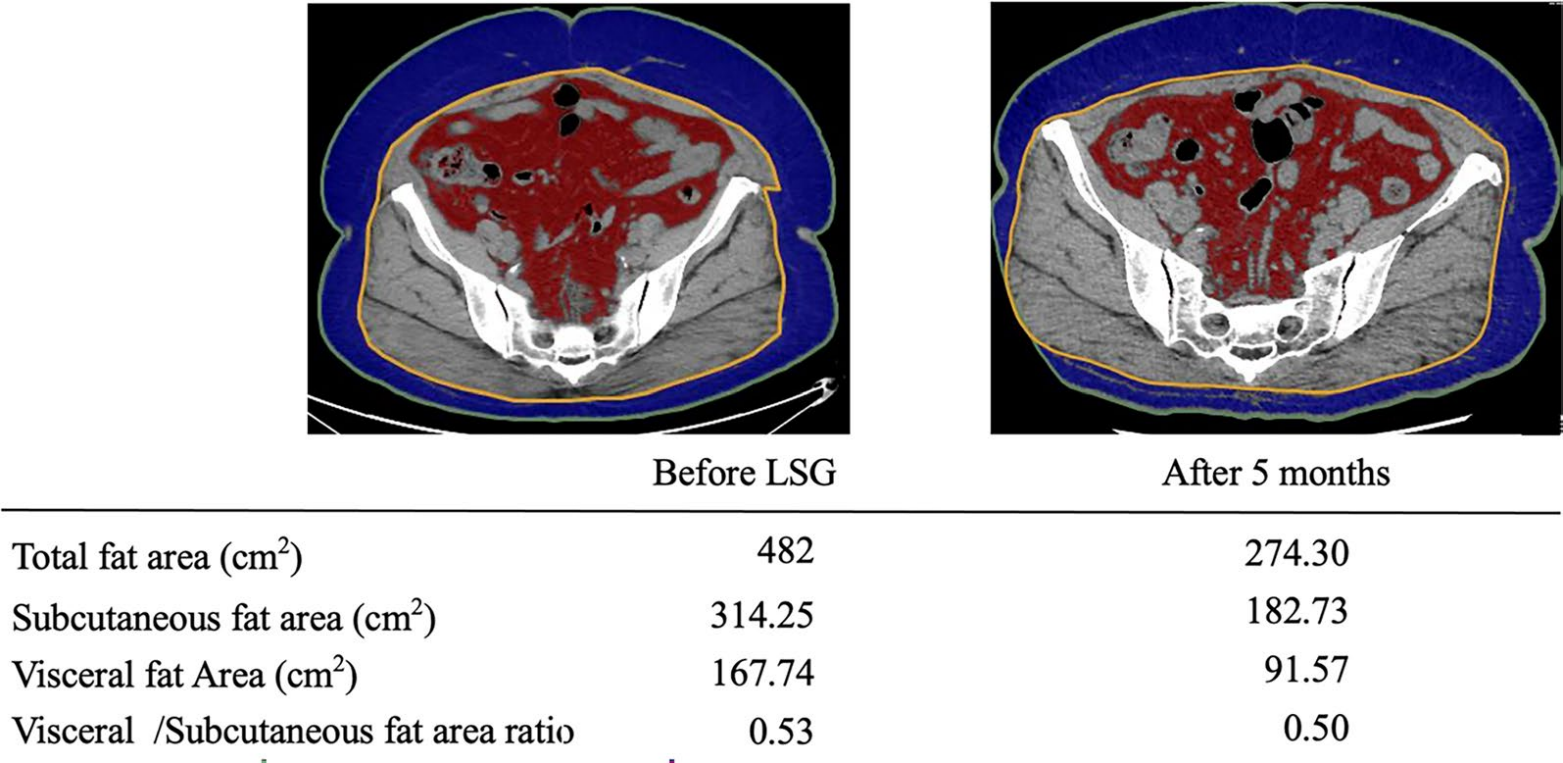
The patient’s trend in body weight and visceral fat area. The table shows the trend in body weight and visceral fat area, and the images show the change in visceral fat area at the level of the umbilicus

LSG was performed using a standardized surgical technique with a 37.5-Fr bougie without any complications. The operation time was 100 min, and blood loss was minimal. She was discharged three days after surgery. Endoscopy showed no change in tumor morphology or size at five months after the LSG. The trends in her change in body weight and visceral fat area at the level of the umbilicus are shown in Fig. 2. Her body weight decreased from 97.0 kg (BMI 40.4 kg/m^2^) to 66.0 kg (BMI 27.8 kg/m^2^) over six months. Visceral fat decreased by 45.4%, and the percent of excess weight loss was 82.3%. Her hypertension went into remission, and she no longer required medication.

## Surgical procedure

**Fig. 3:**
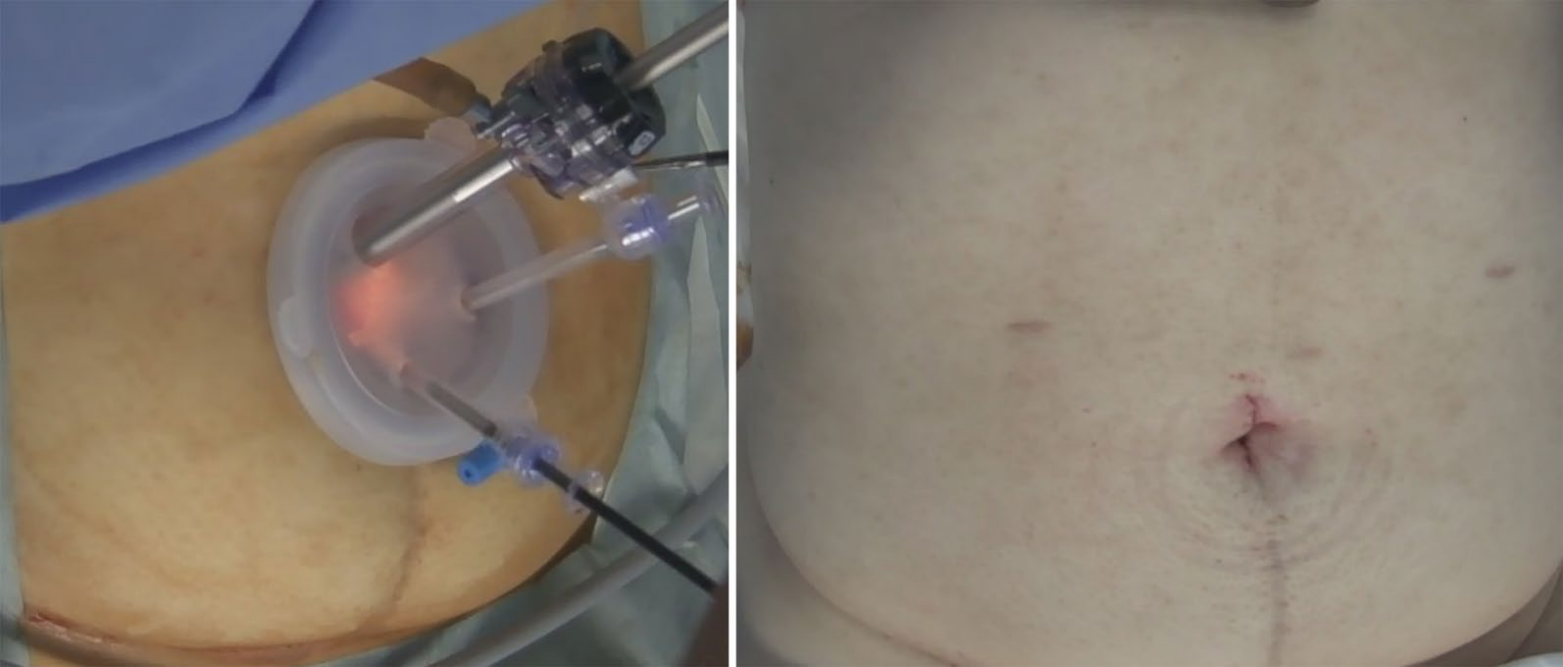
Intraoperative photographs show ileocecal resection via single-incision laparoscopic surgery and the surgical wound postoperatively

**Fig. 4:**
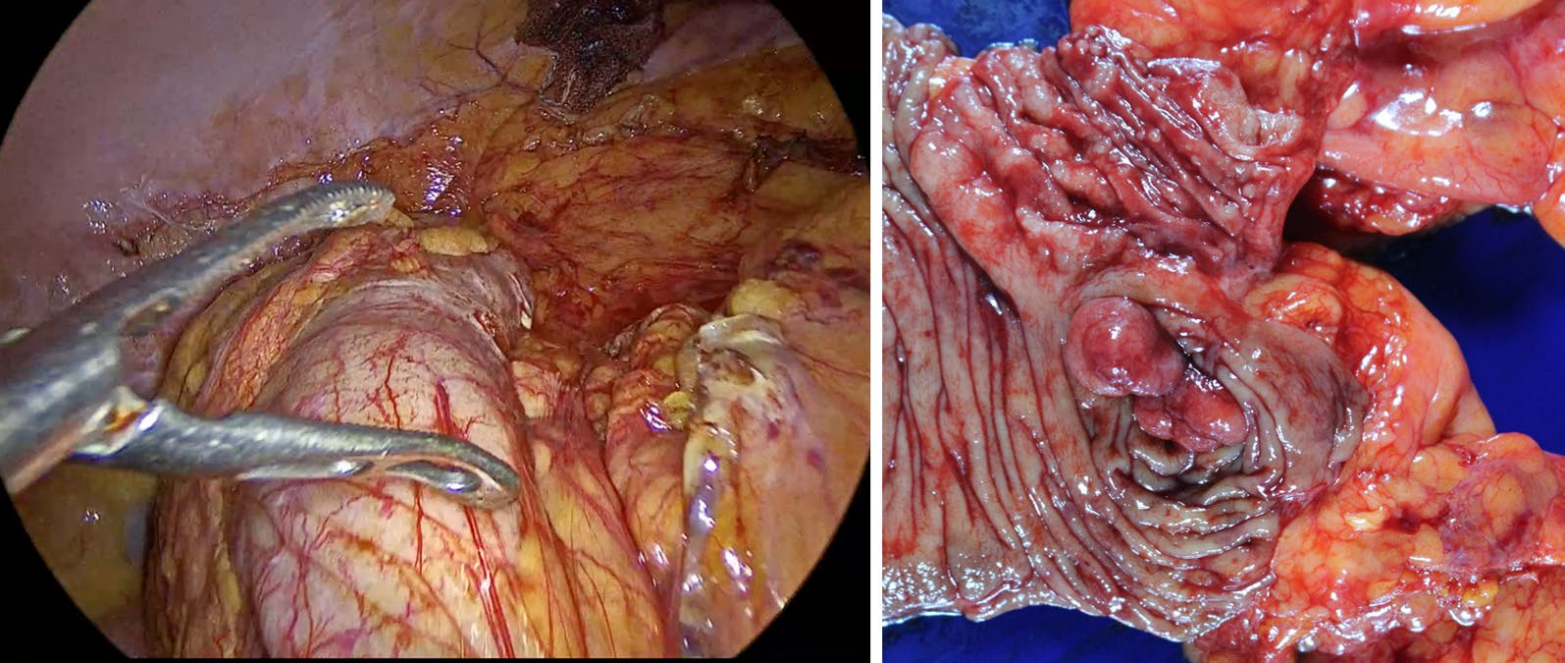
Intraoperative photographs show findings after easy dissection (left panel) and the resected specimen (right panel)

We then performed ileocecal resection and lymph node dissection via single-incision laparoscopic surgery (Fig. 3). The operation was performed via a 3-cm longitudinal incision at the umbilicus. A 12-mm camera port and two 5-mm ports were inserted through an E・Z Access device (Hakko, Nagano, Japan). Mesenteric mobilization was started on the anterior surface of the duodenum (Fig. 4). The ileocolic artery and vein were exposed and dissected (D2 lymph node dissection), and the colon was dissected for tumor resection. We then performed a functional end-to-end anastomosis. The operation time was 108 min, and blood loss was minimal. The number of lymph nodes harvested was 29. She was discharged seven days after surgery without any complications. The pathological diagnosis was adenocarcinoma in adenoma, TisN0M0, pStage 0.

## Discussion

Severe obesity significantly affects the difficulty of colorectal cancer surgery [2]. The increased difficulty of surgical procedures in obese patients has been attributed to the narrow free space in the abdominal cavity, difficulty in excluding the small intestine from the field of view, the weight of heavy organs, tissue fragility, and difficulty in understanding the anatomy of the blood vessels and nerves.

Previous studies have reported that the degree of visceral fat is related to surgery time and the incidence of surgical complications such as surgical site infection and anastomotic failure [5], and fewer lymph nodes are harvested [6]. Visceral fat area is a more useful parameter than BMI in predicting surgical outcomes after laparoscopic colorectal surgery [5].

After LSG, visceral fat decreases in correlation with the amount of weight loss achieved [7]. The decrease in visceral fat after LSG allows for more free space in the abdominal cavity and also facilitates expansion of the visual field, making for safer cancer surgery.

The 2022 ASMBS and IFSO guidelines describe bariatric surgery as a bridge procedure for joint replacement, abdominal wall hernia repair, and organ transplantation [8]. However, these guidelines do not describe bridge surgery for oncologic surgery, and there are a few papers describing such cases.

Gianos et al. reported LSG as a bridge surgery in one case of small bowel carcinoma, two cases of renal tumors, and one case of prostate cancer [9]. Iwata et al. performed LSG followed by thoracoscopic radical surgery for lung cancer detected preoperatively before LSG [10], and Chemaly et al. performed LSG as a bridge procedure in five patients with cancer and severe obesity [11]. Performance of bariatric surgery prior to malignant tumor resection is controversial in terms of disease progression. There is no literature on how long an interval is acceptable for colorectal cancer. Therefore, there are no definitive inclusion criteria for performing bariatric surgery prior to primary tumor resection.

The preoperative clinical diagnosis in this case was tubular adenoma or very early-stage cancer, and resection was indicated. LSG was performed prior to oncologic surgery to reduce the difficulty of the procedure and the possibility of perioperative complications due to obesity. There are currently no recommended criteria for the timing of surgery after LSG. In the 10 patients in the three papers presented here, most underwent oncologic surgery between three and six months after LSG [9–11]. Their mean BMI at baseline was 47.2 kg/m^2^ and that before tumor surgery was 37.3 kg/m^2^ [9–11]. In our patient, LSG also resulted in good weight loss. Because weight loss after LSG is generally rapid up to six months later, we recommend oncologic surgery six months after LSG for early-stage cancer if time permits [12]. Regarding the application of LSG to advanced cancer, it may be considered for cases in which it is possible to wait three to six months due to neoadjuvant therapy before resection of the primary tumor. Safe and reliable D3 dissection may be expected with visceral fat reduction.

Obesity is known to be associated with a high risk of developing many cancers, and the risk of developing colorectal cancer is reported to correlate with obesity status and visceral fat mass [13]. In Japan, the incidence of colorectal cancer is increasing, and it is now the leading cause of cancer-related death in women and the second leading cause of death in men [14]. Therefore, the number of cases requiring bridge surgery may increase, and it will be necessary to discuss the effectiveness of surgery after LSG and the timing of surgery based on the accumulation of more cases in the future. It has also been reported that bariatric surgery reduces the risk of cancer, including colorectal cancer, and LSG as a bridge surgery may be effective in reducing the risk of new colorectal cancers in the future [15].

## Conclusion

LSG was effective in achieving preoperative weight loss before colorectal surgery in a severely obese patient by reducing visceral fat and enabling a safe surgery.

## Data Availability

The data that support the findings of this study are openly available from the corresponding author upon reasonable request.

## Abbreviations

BMI: Body mass index
LSG: Laparoscopic sleeve gastrectomy
